# Integrating evidence-based paediatric asthma management in Australian primary care: phase I protocol for developing implementation bundles

**DOI:** 10.3389/frhs.2026.1723215

**Published:** 2026-03-11

**Authors:** Ashraful Kabir, Md Ariful Islam, Shirdhya Joypaul, Duha Gide, Gaston Arnolda, Yvonne Zurynski, Georgia Fisher, Charlotte Kelly, Yvonne Mullins, Bronwyn Gould, Anthony Flynn, Sinead Burke, Ai-Vee Chua, Charlotte Dealhoy, Christina Rojas, Jeffrey Braithwaite, Adam Jaffe, Nusrat Homaira

**Affiliations:** 1 Discipline of Paediatrics and Child Health, School of Clinical Medicine, Faculty of Medicine, UNSW Sydney, Randwick, NSW, Australia; 2 Australian Institute of Health Innovation, Macquarie University, Sydney, NSW, Australia; 3 Sydney Children’s Hospital, Randwick, NSW, Australia; 4 General Practitioner, Paddington, NSW, Australia; 5 Asthma Australia, Chatswood, NSW, Australia; 6 Practice Nurse, Sydney, NSW, Australia; 7 School of Rural Health, Faculty of Medicine and Health, The University of Sydney, Sydney, NSW, Australia; 8 Western NSW Primary Health Network, Sydney, NSW, Australia; 9 Independent Parent Representative, Sydney, NSW, Australia; 10 MQ Health, Macquarie University, Sydney, NSW, Australia; 11 James P. Grant School of Public Health, BRAC University, Dhaka, Bangladesh

**Keywords:** evidence-based paediatric practice, implementation bundle, implementation science, mixed-methods research, paediatric asthma, primary care

## Abstract

**Introduction:**

Asthma is the most common chronic respiratory condition among Australian children. However, adherence to clinical guidelines for paediatric asthma care in general practice (GP) settings requires attention—it is estimated to be below 60% in some contexts. The National Paediatric Applied Research Translation Initiative (N-PARTI) is a three-phased, co-designed research program aiming to optimise guideline-concordant paediatric care across three priority conditions, including asthma, Type 1 Diabetes (T1D), and antibiotic stewardship in Australian general practices. This protocol outlines Phase I of the N-PARTI asthma stream, focusing on developing an Implementation Bundle to support evidence-based asthma management in general practices.

**Methods and analysis:**

Using a mixed-method design, Phase I will employ a multi-method, co-design approach comprising three Aims: (i) to verify and refine a multicomponent asthma Implementation Bundle tailored for general practice through evidence synthesis, and co-design workshops, involving children with asthma and their parents and carers, alongside with key stakeholders; (ii) to map asthma-related clinical workflows across diverse general practice settings through interviews and observations, analysed using the Functional Resonance Analysis Method (FRAM) to capture variations in routine practice; and (iii) to explore contextual factors within Primary Health Networks (PHNs) through stakeholder interviews, informing the development of locally tailored implementation strategies. Qualitative data will be analysed using a reflexive thematic analysis approach informed by the Consolidated Framework for Implementation Research (CFIR). Outputs will include a refined, contextually adapted paediatric asthma Implementation Bundle and resources to support real-world simulation, testing and tailoring (Phase II), as well as the scale-up, embedding and evaluation of the implementation (Phase III).

**Ethics and dissemination:**

This research project has been approved by the Macquarie University Human Research Ethics Committee (Reference No. 520251855660911). Findings will be disseminated through peer-reviewed publications, conferences, stakeholder forums, and policy briefings. Co-designed outputs will also be shared with participating PHNs to inform wider implementation and scale-up efforts.

## 1 Introduction

Asthma is a major non-communicable disease, affecting more than 262 million people of all ages worldwide and contributing to an estimated 455,000 attributable deaths annually (1). In Australia, it is the most common chronic respiratory condition among children, with 8.2% of those aged 0–14 years reporting a current asthma diagnosis (2, 3). Given the public health significance, Asthma Australia, a peak body, recently identified the need to better address the causes of attacks, treatment, and impacts in children as its top research priority (4).

In 2022–23, approximately 13,500 Australian children aged 14 or under were hospitalised due to asthma (5), with more than 20% of them re-hospitalised within 12 months of their initial admission (6). Frequent hospital presentations due to asthma are a marker of poorly controlled disease, highlighting the need for a more intensive focus on primary care management and post-discharge follow-up within the general practice settings. Evidence from systematic reviews suggests that multi-modal interventions, such as interactive caregiver education tailored to the child's asthma, reduction of home environmental triggers, and regular follow-up, can reduce asthma-related hospitalisations (7). However, caregiver education is time-consuming, intensive, and challenging to implement in acute care settings. These settings typically focus on managing asthma exacerbations and recommend post-discharge assessment with general practitioners (GPs) and their care team (8).

Importantly, the burden of paediatric asthma is not evenly distributed. Recent Australian research has highlighted significant geographic and socioeconomic variation in childhood asthma prevalence, with higher rates clustered in disadvantaged and regional areas, indicating reduced access to care (9, 10). Moreover, children from socioeconomically disadvantaged, Aboriginal and Torres Strait Islander, and culturally and linguistically diverse (CALD) communities exhibit disproportionately higher asthma-related morbidity and hospital presentations compared with the general population (9, 11). Within Australia's multi-level healthcare governance system, regional variation in primary care capacity may further impede the consistent implementation of evidence-based asthma care. This underscores the importance of locally adaptable implementation strategies aligned with community and system contexts (12).

GPs serve as the first point-of-care for providing evidence-based asthma management for children and play a crucial role in ongoing management. In our previous, internationally recognised CareTrack Kids study (13), we reported that guideline-concordant care for paediatric asthma was provided in 54.4% of primary care settings. Key areas for improving paediatric asthma care in primary care include better documentation of asthma triggers (54.9% adherence), more consistent assessment of inhaler technique (20.3% adherence), and increased use of written asthma action plans (46.5% adherence)—all essential for effective asthma management (14).

Addressing these challenges requires a more comprehensive approach than simply offering isolated training for healthcare professionals. Conventional interventions—such as passive dissemination of standard guidelines, one-off educational sessions, or the provision of asthma resources without structured follow-up or systematic support—are commonly described as implementation strategies. However, evidence indicates that these approaches alone are often insufficient to produce sustained practice change or improvements in implementation outcomes such as adoption and fidelity, as shown in implementation studies in diverse healthcare settings (15–17). Instead, a systematic approach to strengthening the delivery of evidence-based care in general practice settings is needed; one that is carefully co-designed with all stakeholders, especially frontline clinicians, patients, and their families. This approach contrasts with traditional approaches that rely on increased training across-the-board for all clinicians or on an intervention study in a targeted group with a control group comparator. Evidence suggests that training programs alone are insufficient (18), and tightly controlled clinical trials cannot simply be applied to complex healthcare environments (19, 20).

In this study, an Implementation Bundle is conceptualised as a purposeful and theory-informed set of complementary implementation strategies designed to operate collectively to address multifaceted, interrelated determinants of practice change within complex healthcare environments. Unlike single strategies that typically target discrete barriers, bundled approaches facilitate sustained implementation of evidence-based care through coordinated, multi-level interventions that involve organisations, providers, and patients. However, existing implementation science literature indicates that structured approaches to selecting and tailoring such strategies remain limited, underscoring the importance of determinant-informed, stakeholder-engaged bundling approaches (21).

To overcome these challenges, the National Paediatric Applied Research Translation Initiative (N-PARTI), a three-phased bespoke research solution, aims to design and deliver a science-informed Implementation Bundle within primary care across three identified priority conditions: asthma, antibiotic prescribing, and type 1 diabetes (T1D) (22). The overarching goals and conceptual framework of N-PARTI have been described previously in a position paper (22). This initiative brings together clinicians, healthcare professionals, policy planners, academics, patients, and families (parents and carers) to co-design, test, and evaluate strategies to improve the delivery of evidence-based care in general practices for Australian children with the three conditions. Nine of the 31 Primary Health Networks (PHNs—regional health administrative bodies to improve primary health care coordination and efficiency across Australia) are partnering in this initiative.

This protocol paper is part of the broader N-PARTI initiative but specifically focuses on asthma. The N-PARTI asthma project will be structured in three phases. Phase I involves co-designing with primary healthcare professionals, consumers (children with asthma and their parents/carers), and PHNs to develop an asthma Implementation Bundle tailored for general practice settings. Phase II will focus on real-world simulation, testing, and iterative refinement of the Bundle. This will include high-fidelity lab-based simulation, followed by field testing in diverse practice settings, supported by Specialist Implementation Teams to identify barriers, enablers, and implementation requirements. Outcomes from Phase II will result in fully field-tested and integrated Implementation Bundles suitable for a wide range of Australian clinical contexts, and modifiable for potential international use. Phase III will focus on scaling up, embedding, and evaluating the Bundles across PHNs nationally, including monitoring outcomes, identifying sustainability factors, and leveraging leadership, clinical champions, and national networks to facilitate adoption and long-term implementation (22). This paper describes the Phase I protocol, which involves developing an Implementation Bundle that can be adapted to increase guideline-concordant paediatric asthma care within varied general practice settings (e.g., rural vs. urban, corporate vs. small-business, with or without practice nurses or nurse practitioners).

### 1.1 Overview of Phase I

**Figure 1:**
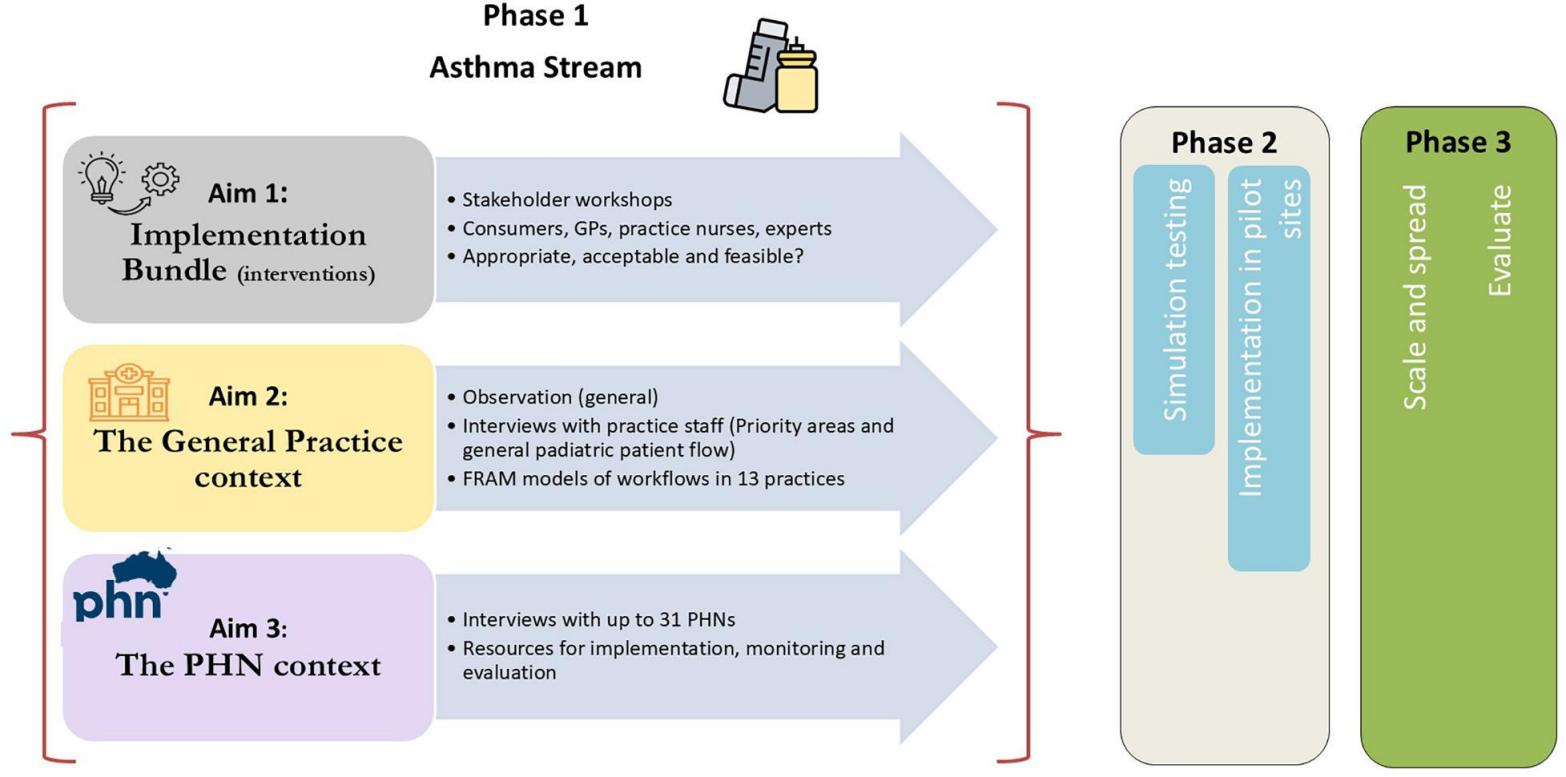
Overarching project translational model for phase I of the asthma stream. Adapted from the N-PARTI position paper (22). GPs, general practitioners; FRAM, functional resonance analysis method; PHNs, Primary Health Networks.

Phase I will commence with the establishment of an asthma steering group to provide strategic oversight of the project's implementation. This phase comprises three specific aims (Figure 1): (i) To verify and refine the asthma Implementation Bundle appropriate for the Australian general practice context, drawing both on previous and concurrent review; (ii) To map clinical workflows within general practice settings; and (iii) To explore the Australian PHN contexts. At its heart is a co-development process involving children with asthma, their parents and carers, GPs, practice nurses, health administrators, PHNs representatives, implementation scientists, and researchers—including those affiliated with peak bodies such as Asthma Australia—and other relevant stakeholders. The outputs of this process will be documented and published upon completion of Phase I. The duration of the co-development process will depend on the availability of PHN staff; however, we aim to complete Phase I within a year of commencement.

## 2 Establishing the asthma steering group

**Figure 2:**
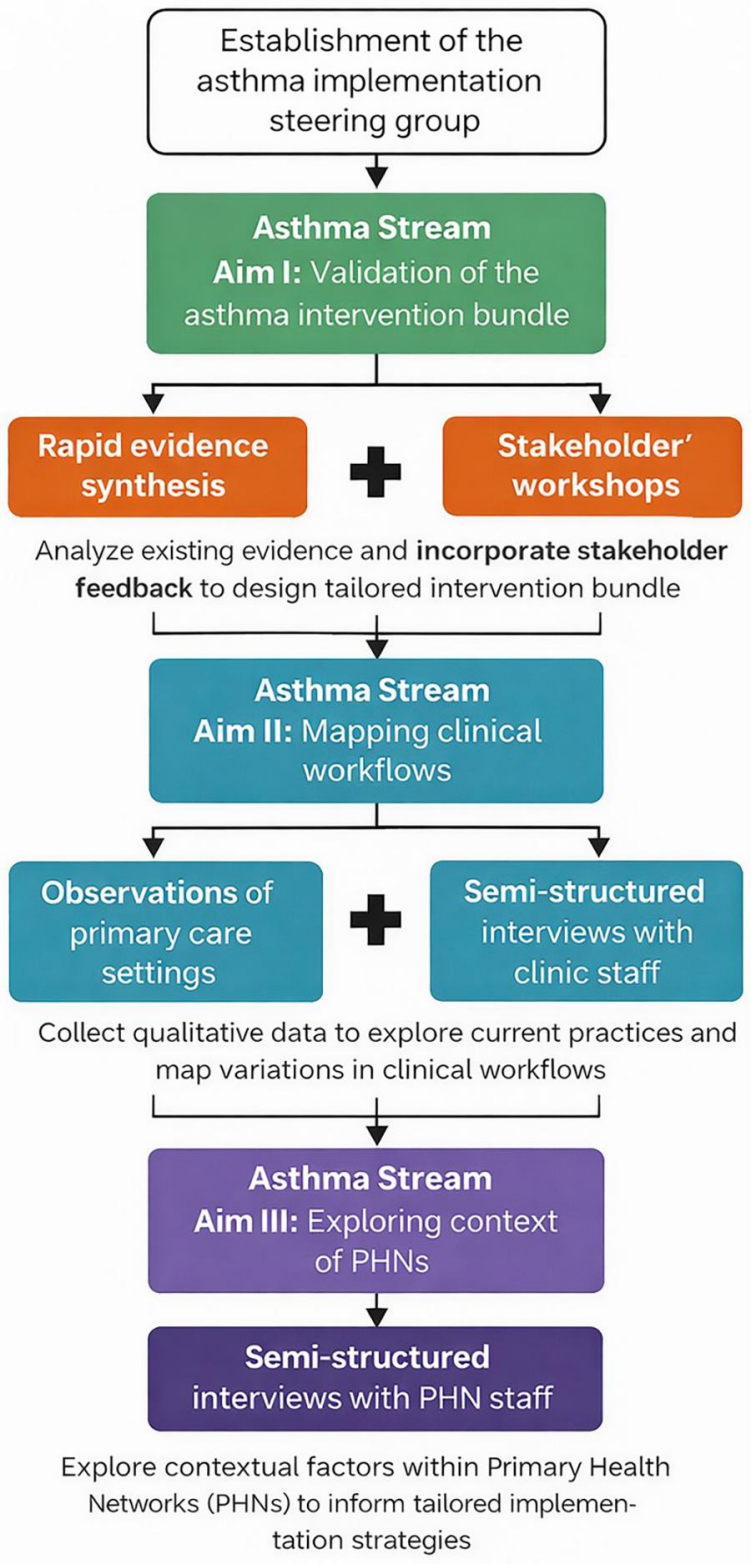
Steps, components, and methods for the three aims of phase I of the asthma stream. PHNs, Primary Health Networks.

The first step of Phase I will involve establishing a steering group to oversee the asthma condition and ensure a comprehensive and collaborative approach (Figure 2). This group will comprise a subset of the broader research team and will include GPs, nurses, researchers, selected PHN representatives, and paediatricians, including respiratory specialists, who will contribute clinical expertise as well as parents/carers. In Australia, PHNs are government-funded, not-for-profit organisations that work towards identifying local community needs in primary care. Strategically, PHNs are well-positioned to conduct empirical investigations into the management of chronic conditions and to provide evidence-based recommendations for ongoing care, thereby reducing patients' need for secondary or tertiary services (23). Our nine partner PHNs represent over 25% of the country. Consumer representatives with experience in paediatric asthma management will be invited through the N-PARTI team's professional networks. The consumer representatives will also be sourced through: (i) the Consumers Health Forum of Australia (24); (ii) Health Consumers NSW (25); and (iii) key asthma organisations, including Asthma Australia (26), the National Asthma Council Australia (27), the Thoracic Society of Australia and New Zealand, and the Centre of Excellence in Severe Asthma (28). Nurse practitioners working in primary care are also envisaged as part of the steering group and will also be recruited through our professional networks. In line with the Health Consumers NSW guidelines (29), and our University Human Research Ethics practices, consumer representatives will be remunerated for their time and collaboration with the steering group. Other participants, such as the health professionals and researchers, will contribute in a professional or salaried capacity, consistent with current institutional practice.

Based on evolving research needs, the steering group may also consider involving additional members—such as postdoctoral researchers, implementation scientists, administrative staff, research officers, or research students—to provide operational support. These considerations will be discussed during the steering group's meetings and may involve the permanent, part-time, or casual engagement, as appropriate. Stakeholder input, along with data generated across Aims 1–3, will be synthesised through structured decision-making processes led by the asthma steering group. Divergent perspectives will be addressed through consensus discussions, data triangulation, and evaluation against predefined implementation criteria, including feasibility, contextual fit, and evidence strength.

## 3 Aim 1

Development, verification and refinement of the asthma Implementation Bundle for Australian primary care.

### 3.1 Aim 1 A: earlier development and verification of the asthma implementation bundle within the hospital settings

Our previous systematic review and meta-analysis of the global evidence base demonstrated that a multicomponent asthma care model, which includes active care coordination to connect patients/families with acute and primary care services, can reduce asthma hospital presentations by 80% (7). Subsequently, in consultation with clinicians, GPs, nurses, parents/families of children with asthma, and industry partners, we piloted asthma quality improvement initiatives at Sydney Children's Hospital (SCH). These initiatives included a co-designed asthma intervention package comprised: (i) an asthma discharge pack containing individualised asthma action plans, follow-up letters for the child's GP and school/childcare, and access to online asthma educational sessions and resources to standardise care post-discharge; (ii) a letter to the GP outlining the recent hospital visit and highlighting best practice for paediatric asthma management. This letter also encouraged the GP to review the child's asthma action plan, assess the need for preventer medication, evaluate the inhaler techniques, and consider referral to a paediatrician if required; (iii) a reminder text message to parents to follow-up visit to their child's GP, reinforce the importance of reviewing the asthma action plan and encourage participation in free asthma education sessions; and (iv) an asthma educational webinar was offered to GPs to support the adoption of best-practice paediatric asthma care (30). Post-implementation data revealed a 60% reduction in hospital presentations among children who previously presented frequently to the SCH. Frequent presentations were defined as four or more visits to the SCH emergency department within 12 months, with at least three of these presentations related to asthma (30).

### 3.2 Aim 1 B: adaptation of the previous SCH asthma implementation bundle to the primary care settings

Collectively, our previous review and SCH research initiatives have informed the design of an asthma Implementation Bundle, including patient-level interventions (provision of standardised asthma resource pack) and practice- and provider-level interventions (through integration of HealthPathways®—a web-based clinical decision support and referral guidance designed to support GPs during consultation and continued professional development courses), specifically targeted for primary care (31). The interventions at different levels are outlined in Table 1. Transitioning the SCH hospital-based bundle to primary care settings will follow a structured, multi-step process. First, we will conduct a rapid review (Aim 1C) to identify evidence-based asthma interventions and implementation strategies used in primary care settings. Findings from this review will be mapped against the existing SCH hospital-based bundle to identify overlapping components, gaps, and areas requiring adaptation. Second, the bundle components will be assessed against predefined criteria, such as relevance to routine general practice workflows, feasibility within standard consultation times, compatibility with existing primary care systems (e.g., HealthPathways®), and acceptability to clinicians and families. Third, components will be retained, modified, or excluded based on triangulation of evidence from the rapid review, stakeholder feedback, and assessment of contextual fit within general practice settings. This structured approach will enhance the transparency and reproducibility of the bundle adaptation process.

**Table 1:** Implementation of asthma intervention for phase I: practice, provider, and patient approaches.

Intervention Level	Description
Practice-level interventions	Integration of asthma HealthPathways® into primary care computing systems and clinical software to enhance accessibility of standardised asthma management guidelines.
Provider-level interventions	Free online module on paediatric asthma management for GPs via ThinkGP—a national web-based educational program providing accredited professional development courses for GPs and other primary care providers across Australia.
	Co-designed webinars will be developed for GPs and practice nurses by respiratory paediatricians and PHN GP leaders, and the provision of written Current Paediatric Asthma Management Practice Points.
Patient-level interventions	An asthma pack for children and families, including individual asthma action plan, QR codes for inhaler technique videos in multiple languages, factsheets, asthma-related resources, school and childcare support materials, and
	Co-designed informational brochure for parents and families at primary care centres.

GP, general practitioner; PHN, Primary Health Network; QR, Quick Response; ThinkGP, national web-based educational platform for accredited GP education; HealthPathways®, web-based clinical information portal providing locally agreed primary care clinical guidance.

The interventions will be further refined through a rapid review of existing evidence to ensure that they are appropriate for the Australian primary care setting as follows:

### 3.3 Aim 1 C: rapid review to verify the proposed asthma implementation bundle for the primary care settings

**Aim:** To identify, synthesise, and summarise evidence on effective Implementation Bundling and strategies for asthma management in primary care settings, applicable to the Australian setting, with a focus on improving guideline-concordant care.

**Method:** Databases including PubMed, Ovid Embase, Ovid PsycInfo, CINAHL, and Cochrane Library will be searched using a combination of keywords and MeSH terms such as “asthma,” “primary care,” “general practice,” “management,” and “intervention,”. The search strategy will also include implementation-focused terms such as “implementation,” “implementation strategy,” “quality improvement,” and “practice change.” The strategy will be refined in consultation with an academic librarian. Two reviewers will independently screen titles and abstracts, followed by full-text screening of potentially relevant studies. Discrepancies will be resolved through discussion or the involvement of a third reviewer. A standardised form will be used to extract data on study characteristics (author, year, country, design), population and setting, intervention details, outcomes, and key findings of the included studies.

**Data analysis**: Findings will be synthesised narratively and grouped by intervention type. Where feasible, a summary table will be developed to present key characteristics of the included studies, including their reported effectiveness (e.g., improved asthma control, reduced hospitalisation or emergency visits), implementation logistics (e.g., training requirements, delivery settings), and relevance to Australian primary care, particularly in terms of implications for clinical workflows and patient engagement. Where reported, information on study design and methodological quality will be extracted, enabling the evaluation of the strength of the evidence when prioritising intervention components for inclusion in the Implementation Bundle.

**Outputs:** In addition to publishing the rapid review findings, a key output will be the categorisation of interventions with reported effectiveness in supporting guideline-concordant asthma care in primary care settings. For each identified intervention category, a one-page summary will be developed to inform the subsequent evidence verification process. These summaries will draw on the findings from the data analysis and outline effectiveness, implementation logistics, and implications for the integration within Australian general practice.

### 3.4 Aim 1D: evidence refinement through stakeholder workshops

**Aim:** To assess the acceptability, feasibility and appropriateness of the interventions identified in the rapid review, specifically for use in the Australian general practice context, as perceived by clinicians, consumers and PHNs.

**Design:** Interactive stakeholder workshops.

**Participants:** Approximately 15–20, including clinicians, practice nurses, PHN representatives, paediatricians, consumers (children with asthma and their parents and carers), and other relevant primary care staff. Participants will be drawn from a wide range of perspectives, geographical locations and contexts to ensure maximum diversity.

**Recruitment:** Participants will be recruited purposively through established professional networks and peak bodies, such as Asthma Australia, the Royal Australian College of General Practitioners (RACGP), the Australian Primary Healthcare Nurses Association (APNA), and nine partner PHNs. Consumers will be recruited through the Consumers' Health Forum of Australia, Health Consumers NSW, or via professional networks of the steering group members. To acknowledge their time and contributions, consumers will receive remuneration for their participation, while GPs and practice nurses will be offered gift vouchers, as is consistent with standard institutional practice.

**Method:** A round of structured workshops will be conducted to review the Implementation Bundle. The workshop will be facilitated by a qualitative researcher in collaboration with the asthma stream implementation science team. Each workshop will consist of 8–12 participants and last 1–3 h, depending on the complexity of the topic, the number of discussion points, and the feedback required (32). The session will focus on the practicability, clinical relevance, health system context, and usability of each component of the Implementation Bundle within general practice settings. Activities will be designed to stimulate co-creation with children and parents/carers, ensuring that lived experience informs refinement of the bundle. Participants will review a summary of the Implementation components drawn from previous research and current rapid review findings. Qualitative data collection tools, including workshop guides and prompts, will be informed by key domains of the Consolidated Framework for Implementation Research (CFIR), including Intervention Characteristics, Inner Setting, Outer Setting, Characteristics of Individuals, and Process. These domains will guide both data collection and the initial coding framework to support systematic analysis of implementation determinants (33, 34). Paediatricians, including respiratory specialists, will contribute their clinical expertise during workshops to support the development, refinement, and contextualisation of the Implementation Bundle. The subsequent discussions will focus on real-world applications, contextual factors within the general practices, and anticipated barriers and enablers to implementation. Additional workshops with the same stakeholder groups or other relevant stakeholders may be considered as research progresses.

**Data analysis:** The stakeholders' workshop will primarily generate qualitative data from multiple sources, including recordings of the group discussions, open discussions, workshop notes, written feedback and comments, and outputs from group work. Data analysis will focus on stakeholders' views and perspectives on the relevance, acceptability, and feasibility of the proposed Implementation Bundle, as well as on refining and validating its components. A rapid qualitative analysis approach will be used to analyse data. This approach is increasingly used in implementation and health service research, enabling quick, rigorous, and synthesised analysis for policy, practice, and decision-making (35, 36). A matrix-based approach will be designed and implemented to integrate all data types and generate a structured summary of the discussion for each topic or component of the Implementation Bundle, based on preliminary codes derived from the moderator guide (37). The summary and preliminary code will be mapped into the data matrix, organised by barriers and enablers of the proposed Implementation Bundle. The analysis team will review the matrix to refine the coding, group the themes into meaningful categories, and compare them across barriers and enablers (36, 37).

**Outputs:** Findings from the workshops will inform refinement of the Implementation Bundle. Phase I will focus on assessing implementation outcomes, including acceptability, feasibility, and appropriateness, as defined in established implementation outcome frameworks. These findings will guide the refinement of Implementation Bundle components and inform decisions about the progression to simulation testing in Phase II. The steering group will review a summary of the feedback to inform decisions on the final components of the Implementation Bundle, which will be advanced for piloting in the subsequent phases of N-PARTI. The refined Implementation Bundle will be designed to allow flexible adaptation, ensuring alignment with practice needs and preferences across different general practice settings. A preliminary theory-informed logic model will be developed to articulate hypothesised pathways through which bundle components influence implementation determinants and anticipated outcomes. For example, clinician education modules are expected to enhance knowledge and self-efficacy; workflow integration strategies are aimed at reducing organisational barriers; and patient-focused resources are intended to improve engagement and adherence. Components of the bundle will be iteratively reviewed and refined, based on stakeholder feedback, implementation determinants analysis, and feasibility assessments, during subsequent phases to minimise the risk that the bundle functions as a black-box intervention.

Bundle refinement will systematically incorporate equity-related factors through purposive sampling and targeted stakeholder engagement. Specifically, workshops will include representation from rural and regional PHNs and practices serving rural and remote populations, as well as practices with a high proportion of culturally and linguistically diverse (CALD) patients. Consumer participants will be recruited to reflect linguistic and cultural diversity, and materials will be reviewed for cultural safety and language accessibility. Equity considerations, including barriers related to geography, workforce capacity, and language, will be explicitly explored during workshops and incorporated into bundle refinement to enhance applicability across diverse populations and practice contexts. As part of the refinement process, bundle components will be categorised as either core elements, which are essential for maintaining intervention integrity and expected mechanisms of action, or adaptable elements that may be tailored to local contexts, workforce resources, and patient needs. This classification will support scalability, fidelity monitoring, and enhance transferability across diverse primary care settings.

## 4 Aim 2: accurately map clinical workflows in primary care settings

In 2024, there were approximately 7,135 accredited general practices in Australia (38). Based on an estimated 80% accreditation rate, the total number of general practices is around 9,000 (38). Our internal analysis of 2018 data from the Medicine in Australia: Balancing Employment and Life (MABEL) survey identifies key characteristics of these practices, including location [classified according to the Australian Standard Geographical Classification (ASGC)], corporate ownership, and the average staffing profile, such as the number of doctors and nurses per practice (39). Approximately 70% of practices are located in metropolitan areas, 20% in inner regional areas, and 10% in outer regional, remote, and very remote locations. Around one-third of the practices are corporate-owned. Staffing varies across regions. The median number of GPs per practice is eight in metropolitan and inner regional areas, and six in more rural locations (41). Most practices employ nurses, with a median of three nurses across all locations, though remote practices are more likely to have six or more nurses (20% compared to 10% in major cities). Notably, over 20% of MABEL respondents reported having advanced skills in paediatrics or adolescent health (41).

Aims: To map existing workflows within the general practice settings, specifically relating to paediatric asthma care, and to identify how the proposed asthma Implementation Bundle can be effectively integrated into existing workflows.

**Design:** A mixed-method approach combining non-participant observation and semi-structured interviews will be used to map asthma care workflows in the general practice clinic.

**Participants:** This component will be conducted exclusively within the PHNs partnering in the N-PARTI project. A purposive sample of 13 general practices will be selected across these PHNs to ensure a broad and maximum representation. This will include six metropolitan practices, of which two will be corporate-owned, as well as three inner regional practices, including one corporate-owned. It will also include two remote practices and one practice each from outer regional and very remote areas. To ensure diverse representation, the sample will include at least one practice that serves a high proportion of Aboriginal and Torres Strait Islander patients, and at least one that serves culturally and linguistically diverse (CALD) populations. The perspectives of Indigenous and CALD consumers will be incorporated into the design, analysis, and interpretation to ensure cultural appropriateness and relevance. This will be achieved through the purposive recruitment of participants from these communities, consultation with relevant consumer representatives, and the engagement of cultural groups where appropriate. This approach will help ensure that the design of the Implementation Bundle is culturally appropriate, contextually relevant, and responsive to the needs and priorities of this population.

**Recruitment:** General practices will be recruited in consultation with the nine PHN partners. Each participating practice will receive AUD1,000 to compensate for their time and any potential disruption resulting from their participation. Informed consent will be obtained from both practice management and all individual participants. All data will be de-identified, and no practice or staff member will be named in any report or any form of publication.

**Methods:** The study will involve a three-day visit to each participating general practice. During the first two days, non-participant observations will be conducted to capture patient flow and general practice operations. The third day will be dedicated to structured assessments of asthma-related workflows guided by the priorities identified by the asthma steering group. As patients with asthma may not attend the general practice during the observation period, structured interviews will be conducted with staff of the respective general practice to document the existing asthma workflows. Interviews will include one administrative staff member (preferably the practice manager or an experienced receptionist), at least two GPs, and at least one practice nurse or nurse practitioner. Interviews will be conducted in a single session at each general practice. Data collection, including interviews and observations, will be led by the N-PARTI qualitative researcher, with support from Implementation Scientists.

**Data analysis:** Asthma-related workflows at each site's general practice will be analysed and visualised using the Functional Resonance Analysis Method (FRAM). FRAM is a well-established approach for modelling complex socio-technical systems, and offers a nuanced understanding of how clinical work is delivered (40, 41). By integrating the variability, context, and interdependencies in real-world settings, FRAM enables a detailed representation of the current practices. This method ensures that any proposed modifications to workflows or systems are not only realistic and efficient but also aligned with the ways healthcare providers work and responsive to patient expectations (42, 43). Insights from FRAM-based workflow modelling will be mapped onto CFIR domains to identify key implementation determinants. FRAM outputs that capture variability in workflows, resource use, and functional interdependencies will inform CFIR-guided analyses across contextual, individual, and process-related domains, thereby informing the selection and tailoring of strategies within the Implementation Bundle. Where feasible, participating clinic staff will be engaged to review and validate FRAM models, ensuring accuracy and identifying missing steps, adaptations, or contextual influences.

**Outputs:** Synthesised data will help identify broader patterns both within and across general practice settings. This analysis will focus on mapping distinct care pathways and their associated time signatures, which may inform the design and timing of the Implementation Bundle. For example, extended wait times may present opportunities to deliver targeted educational materials, while routine nurse consultations prior to GP visits could serve as key moments to initiate shared decision-making. These insights will directly inform the integration of asthma interventions into the existing workflows during the subsequent phases of the N-PARTI project.

## 5 Aim 3: exploring PHN contexts

There is currently limited evidence base detailing the quality improvement activities undertaken by PHNs, including who implements them, how they are implemented, and the extent to which data is used for monitoring and evaluation. Similar to workflow mapping undertaken in Aim 2, this component of Phase I will inform all three priority streams of the N-PARTI. The details related to the asthma stream are described below.

**Aim:** To document key aspects of PHN operations relevant to N-PARTI, including the use of HealthPathways, involvement in general practices' quality improvement initiatives, and access to and utilisation of practice-level data.

**Design:** Semi-structured virtual interviews will be conducted to explore variations in PHNs' resources, engagement in quality improvement, and access to the data system.

**Participants:** All 31 PHNs will be invited to participate. The process will begin with the nine PHNs partnering in N-PARTI, allowing for refinement of the interview protocol before extending the invitations to the remaining 22 PHNs to participate.

**Recruitment:** PHN Chief Executives will be contacted to provide initial consent and nominate a designated contact person to identify suitable staff to participate in the interviews. The designated contact will facilitate reaching out to potential interviewees. To minimise bias, N-PARTI team members will not be directly involved in participant selection. All interviewees will receive project information and provide informed consent before participating in interviews.

**Methods:** In each participating PHN, two N-PARTI project team members will conduct interviews with up to three to four staff members. Interviews will focus on the current quality improvement activities related to asthma care, including guideline-concordant asthma care and management, use of asthma action plans, and inhaler technique education. Broader system-level issues and contextual factors will also be explored, such as stakeholder roles in asthma-related quality improvement, effective asthma care within the general practices, and the use of data systems. Additionally, the study will examine how PHNs engage GPs through strategies like audit, feedback, and evaluation. This will also explore how PHNs plan and coordinate the rollout of asthma-specific N-PARTI activities alongside existing initiatives, without causing disruption.

**Data analysis:** Interview data will be thematically analysed to identify contextual factors influencing the execution of the Implementation Bundle. These may include workforce composition, service delivery mechanism, and quality improvement infrastructure. NVivo software will be used for coding and analysis. Descriptive analysis of PHN-related data, such as resource availability, models of care, and geographical coverage, will be performed. These findings will be cross-referenced with themes generated in qualitative analysis to inform the development of a tailored Implementation Bundle. This integrative approach will help identify appropriate support tools and strategies for implementation in diverse PHN settings. All data will be de-identified to maintain the confidentiality of both PHNs and individual staff members. Findings from Aim 3 will be integrated with workflow findings from Aim 2 to guide the final refinement and adaptation of the Implementation Bundle at the system and practice levels.

**Output:** An analysis of published literature, as part of a scoping review on PHN operations, as well as an analysis of publicly available documents, such as annual reports, strategic plans, and the Australian Institute of Health and Welfare dataset, will be carried out prior to the interviews. This preparatory work will inform and tailor the interview guide to reflect each PHN's strategic priorities and operational context. This step may also result in a dedicated publication or commentary. Data from the interviews will be subsequently synthesised and submitted for publication as an academic research paper, contributing to the broader evidence base on implementation strategies across diverse PHN contexts. Additionally, a comprehensive internal report will be generated to inform the phased roll-out and continuous improvement of the N-PARTI initiative.

## 6 Outcomes of phase I

Outcomes of Phase I will include a tailored, co-designed Implementation Bundle for asthma care. The bundle will comprise evidence-based implementation tools, practical guides, supporting data systems, and capacity-building resources. At the completion of Phase I, the Implementation Bundle will be specified as a clearly defined and enumerated set of discrete implementation strategies, complemented by supporting tools, operational guidance, and delivery descriptions. While the core components and delivery mechanism will be sufficiently standardised to support transparency, replicability, and structured testing, adaptable elements will allow contextual tailoring to local practice settings.

These outputs will provide the foundation for phase II, involving iterative simulation-based testing and real-world piloting to assess usability, acceptability, and feasibility, and for Phase III, which focuses on national-scale up, embedding, and evaluation with ongoing monitoring and support. The sustainability of the Implementation Bundle will be enhanced by aligning its components with existing PHN quality improvement, programs, professional development pathways, and routine practice workflows, promoting continued use beyond Phase I.

## 7 Ethics considerations

Ethics approval for Phase I components—Aims 1D, 2 and 3 has been obtained from the Macquarie University Human Research Ethics Committee (Reference No. 520251855660911). Ethics approval was not required for Aims 1A (development of previous bundle in hospital setting), 1B (adaptation of previous bundle to primary care setting), 1C (rapid review) or the establishment of the steering group, as these activities do not involve human participants.

## 8 Discussion and conclusion

Asthma is the most common chronic respiratory condition among Australian children, creating a substantial disease burden on individuals, families, and the healthcare system (9). Although effective treatment options and clinical guidelines are available, passive dissemination of these resources might not have led to consistently high levels of guideline-concordant care in primary care settings. There is a pressing need to support the uptake of the best-practice asthma care in ways that are practical and meaningful for both the providers and consumers. This study presents a robust, theory-informed protocol to co-design and refine an evidence-based Implementation Bundle for paediatric asthma care in Australian primary care settings. A key strength of this study is the integration of a theory-driven, participatory, and comprehensive mixed-methods design, which allows for the generation of rich, complementary qualitative and quantitative data. This design provides a holistic understanding of implementation processes within diverse general practice environments. The use of well-established conceptual frameworks—such as the Consolidated Framework for Implementation Research (CFIR) and the Functional Resonance Analysis Method (FRAM)—further strengthens the methodological rigour by enabling systematic exploration of clinical workflows and implementation determinants. This ensures the Implementation Bundle is not only evidence-informed but also contextualised and tailored to real-world practice settings.

Another notable strength lies in the adoption of a participatory approach, which uses codesigning techniques involving a wide range of stakeholders, including clinicians, PHN representatives, consumers, and implementation scientists. This ensures that the bundle will be both acceptable and feasible, and that it addresses barriers and enablers relevant to those involved in paediatric asthma care delivery. Additionally, mapping clinical workflows will help explore contextual barriers and enablers of implementation dynamics.

Despite its strengths, the study's complexity may pose challenges in coordinating data collection and analysis across diverse sites. Variability in stakeholder engagement, particularly among underrepresented groups, could also affect the generalisability of findings. These risks will be addressed through structured engagement and close collaboration with PHNs to support consistent participation. Overall, the co-design process and contextual integration of the Implementation Bundle are anticipated to enhance both its applicability and potential for scale-up across Australian general practices.

## 9 Dissemination strategy

In line with Phase I aims, findings from this study will be disseminated widely to maximise impact and uptake. Outputs will be shared through peer-reviewed publications, conference presentations, and stakeholder forums. Co-designed outputs, including the refined Implementation Bundle and related resources, will be provided to participating Primary Health Networks (PHNs), consumers, and steering committee members to inform broader implementation efforts. Policy briefs will be developed to support translation into practice and guide scale-up strategies. All dissemination activities will prioritise accessibility, relevance, and practical utility for clinicians, consumers, researchers, and policymakers.
